# BHCMDA: A New Biased Heat Conduction Based Method for Potential MiRNA-Disease Association Prediction

**DOI:** 10.3389/fgene.2020.00384

**Published:** 2020-04-28

**Authors:** Xianyou Zhu, Xuzai Wang, Haochen Zhao, Tingrui Pei, Linai Kuang, Lei Wang

**Affiliations:** ^1^College of Computer Science and Technology, Hengyang Normal University, Hengyang, China; ^2^Key Laboratory of Hunan Province for Internet of Things and Information Security, Xiangtan University, Xiangtan, China; ^3^College of Computer Engineering & Applied Mathematics, Changsha University, Changsha, China

**Keywords:** miRNA-disease association, bipartite graph network, biased heat conduction, clustering algorithm, integrated similarity

## Abstract

Recent studies have indicated that microRNAs (miRNAs) are closely related to sundry human sophisticated diseases. According to the surmise that functionally similar miRNAs are more likely associated with phenotypically similar diseases, researchers have proposed a variety of valid computational models through integrating known miRNA-disease associations, disease semantic similarity, miRNA functional similarity, and Gaussian interaction profile kernel similarity to discover the potential miRNA-disease relationships in biomedical researches. Taking account of the limitations of previous computational models, a new computational model based on biased heat conduction for MiRNA-Disease Association prediction (BHCMDA) was proposed in this paper, which can achieve the AUC of 0.8890 in LOOCV (Leave-One-Out Cross Validation) and the mean AUC of 0.9060, 0.8931 under the framework of twofold cross validation, fivefold cross validation, respectively. In addition, BHCMDA was further implemented to the case studies of three vital human cancers, and simulation results illustrated that there were 88% (Esophageal Neoplasms), 92% (Colonic Neoplasms) and 92% (Lymphoma) out of top 50 predicted miRNAs having been confirmed by experimental literatures, separately, which demonstrated the good performance of BHCMDA as well. Thence, BHCMDA would be a useful calculative resource for potential miRNA-disease association prediction.

## Introduction

MicroRNAs (miRNAs) are a class of endogenous regulatory non-coding RNAs found in eukaryotes which are about 20 to 25 nucleotides in length. They were normally considered to be negative gene regulators which suppressed the expression of messenger RNAs (mRNAs) and inhibited the protein translation of target genes (Meister and Tuschl, 2004). However, some studies had confirmed that miRNAs could also play a positive regulatory role (Jopling et al., 2005). In recent years, the studies about the miRNA-disease associations have attracted more and more attentions in consideration of miRNAs having been identified to play a vital role in many important biological processes including cell proliferation, cell development, cell differentiation, cell apoptosis, cell metabolism, cell aging, cell signal transduction, cell viral infection and so on (Xu et al., 2004; Cheng et al., 2005; Miska, 2005; Cui et al., 2006; Bartel, 2009). For example, mir-31 and mir-335 were proved to be effective inhibitors of breast cancer (Tavazoie et al., 2008; Valastyan et al., 2009; Png et al., 2011). miR-122 inhibited cell proliferation and tumorigenesis in certain breast cancer patients by targeting IGF1R (Wang et al., 2012). In addition, researchers discovered that the expression of miR-126 in the blood of patients with Crohn’s disease was significantly higher than normal people (Paraskevi et al., 2012). Moreover, the levels of miR-134 and mir-27b were found to be significantly lower in lung tumors than that in normal tissues, which demonstrated that they were associated with lung cancer (Hirota et al., 2012). Therefore, discovery of disease-related miRNAs is significant for the diagnosis, treatment and prevention of complex human diseases.

Up to now, based on the concept that functionally associated miRNAs are more likely related with phenotypically similar disease, a great number of computational models have been proposed to predict potential associations between diseases and miRNAs. For instance, Jiang et al. (2010) raised a hypergeometric distribution-based computational model through adopting miRNA-target interactions. Shi et al. (2013) developed a computational model by concentrating on the functional interlinkage between diseases and miRNAs and implementing random walk on the protein-protein interaction network. Mork et al. (2014) proposed a computational model called miRPD by integrating protein-disease associations and miRNA–protein associations for prediction of miRNA-Protein-Disease associations. Xuan et al. (2013) presented a computational method named HDMP to infer potential disease-related miRNAs based on weighted *k* most similar neighbors. Chen et al. (2012) developed the global network similarity-based prediction model called RWRMDA by applying random walk to the functional similarity network of miRNA-miRNA to search for potential associations between miRNAs and diseases. However, all these models mentioned above cannot be utilized to predict miRNAs associated new diseases while there are no known miRNA-target associations, since these models rely heavily on known miRNA-target interactions. In recent years, deep learning has been increasingly used to solve many problems, providing an important solution to improve related performance in the field of bioinformatics (Le et al., 2017, 2018). Therefore, in order to solve this problem, Chen and Yan (2014) developed a semi-supervised model called RLSMDA on the basis of regularized least squares, in which negative samples were not required. Zou et al. (2015) introduced two prediction models such as KATZ and CATAPULT to infer potential microRNA-disease associations based on machine learning method. Chen et al. (2016b) put forward a computational model called WBSMDA which was effective for both novel diseases without any known related miRNAs and novel miRNAs without any known associated diseases. Luo et al. (2017) proposed a prediction model named KRLSM to infer potential or missing miRNA-disease associations through integrating miRNA space and disease space into a total miRNA-disease space based on Kronecker product. Chen et al. (2018b) raised a decision tree learning-based model called EGBMMDA, which could serve as a valuable complement to the experimental approach for discovering potential miRNA-disease connections.

**FIGURE 1:**
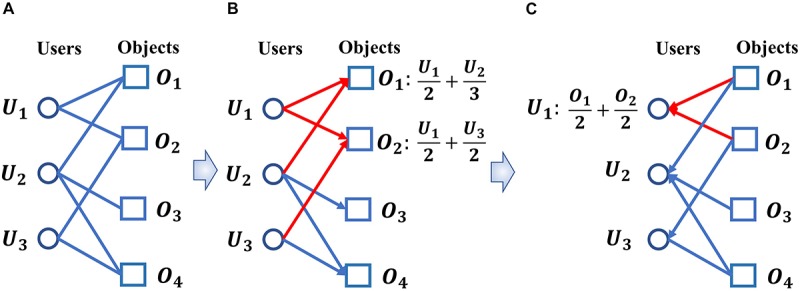
The heat transfer process of biased heat conduction (BHC) algorithm. **(A)** A binary network of users and objects. **(B)** The process of objects receiving resources from users. **(C)** The process of users receiving resources from objects.

Different from above mentioned prediction models, in this paper, a new calculative model called BHCMDA based on Biased heat conduction (BHC) was developed for prediction of potential miRNA-disease association, in which, known miRNA-disease associations, disease semantic similarity, miRNA functional similarity and Gaussian interaction profile kernel similarity were integrated first, and then, the BHC algorithm was adopted to compute both the resources eventually received by miRNAs starting from the miRNA nodes and the resources eventually received by diseases starting from the disease nodes. BHC algorithm is a kind of personalized recommendation algorithm (Liu et al., 2011). Its process is like the transfer of heat in the binary network between the users and the objects. Because the influence of the user’s degree and the object’s degree are considered into the process of heat transfer, the accuracy of recommending the object that the user is interested in is improved. The transfer process is shown in Figure 1. Figure 1A shows a binary network of users and objects. Figure 1B shows the process of object *O*_1_ and object *O*_2_ receiving resources from users. Figure 1C shows the process of user *U*_1_ receiving the resource from the objects. Finally, we averaged these two kinds of resources received by miRNAs and diseases to predict potential miRNA-disease associations. Moreover, in order to evaluate the performance of BHCMDA, twofold cross-validation (twofold CV), fivefold cross-validation (fivefold CV) and leave-one-out cross-validation (LOOCV) were implemented. As a result, BHCMDA could achieve reliable AUCs of 0.8890, 0.9060, and 0.8931 in LOOCV, twofold CV and fivefold CV separately. Furthermore, case studies of esophageal neoplasms, colonic neoplasms and lymphoma were taken to evaluate BHCMDA as well. The simulation results showed that there were 44, 46, and 46 out of top 50 predicted miRNA-disease associations for these three kinds of vital diseases, respectively. Hence, it is obvious that BHCMDA has good performance on prediction of potential miRNA-disease associations.

## Materials and Methods

### MiRNA-Disease Associations

First, we downloaded the known miRNA-disease associations from the HMDD V2.0 database, which consisted of 5430 experimentally verified miRNA-disease associations including 383 diseases and 495 miRNAs (Li et al., 2013). Based on these known miRNAs-disease associations, an adjacency matrix *A* can be obtained according to the following formula:

$$a_{i\,j}=\,\left\{ \begin{array}{c} 1:\mathrm{if}\,\mathrm{there}\,\mathrm{is}\,\mathrm{known}\,\mathrm{assocaitionbetween}\,\mathrm{the}\,\mathrm{miRNA}\,m_{i}\\ \mathrm{and}\,\mathrm{the}\,\mathrm{disease}\,d_{j}\\ 0:\;\mathrm{otherwise}\;\left(1\right) \end{array}\right.$$

### MiRNA Functional Similarity

Moreover, based on the assumption that functionally similar miRNAs are more likely associated with phenotypically similar diseases, the miRNA functional similarity scores can be obtained through adopting the modus put forward by Wang et al. (2010). For simplicity, we downloaded the miRNA functional similarity scores from http://www.cuilab.cn/files/images/cuilab/misim.zip directly and utilized these miRNA functional similarity scores to construct a miRNA functional similarity matrix *FS*, in which, the entity *FS*(*i, j*) indicated the functional similarity between the miRNAs *m*_*i*_ and *m*_*j*_.

### Disease Semantic Similarity Model I

Furthermore, for all these 383 diseases obtained previously, we downloaded their *MeSH* descriptors from the *MeSH* database^1^, and based on these *MeSH* descriptors, each disease *D* could be described by a Directed Acyclic Graph (DAG) such as *DAG*(*D*) = (*D*,*T*(*D*),*E*(*D*)) (Chen, 2015; Chen et al., 2016a; Huang et al., 2016), in which, *T*(*D*) indicated the node set containing node *D* and its ancestor nodes, and *E*(*D*) denoted the edge set involving the direct edges which linked the parent nodes to the child nodes. Hence, based on the concept of *DAG*, the semantic value of the disease *D* could be obtained according to the following formula:

$$D\,V\,1\,\left(D\right)=\sum_{d\in T\,\left(D\right)}D\,1_{D}\,\left(d\right)\;\left(2\right)$$

Here, *D*1*_*D*_*(*d*) represented the contribution of the node d in *T*(*D*) to the semantic value of the disease *D*, which could be obtained according to the following formula:

$$\,\left\{ \begin{array}{c} D\,1_{D}\,\left(d\right)=1\;\mathrm{if}\,d=D\\ D\,1_{D}\,\left(d\right)=\text{max}\,\left\{\Delta \times D\,1_{D}\,\left(d^{\prime }\right)|d^{\prime }\in \mathrm{chiledren}\,\mathrm{of}\,d\right\}\\ \text{if}\,d\neq D \end{array}\right.\;\left(3\right)$$

Here, Δ denoted the semantic contribution factor. From formula (3), it is easy to see that for the disease *D*, its contribution to the semantic value of itself is equal to 1, while for any other disease d in *T*(*D*), as the distance from d to *D* increases, the contribution of *d* to *D* will decrease. Hence, based on the assumption that similar diseases are inclined to share larger parts of their *DAGs*, the semantic similarity between two disease *d*_*i*_ and *d*_*j*_ could be obtained according to the following formula:

$$S\,S\,1\,\left(i,j\right)=\frac{\sum_{t\in T\,\left(d_{i}\right)\,\cap T\,\left(d_{j}\right)}\left(D\,1_{d_{i}}\,\left(t\right)+D\,1_{d_{j}}\,\left(t\right)\right)}{D\,V\,1\,\left(d_{i}\right)+D\,V\,1\,\left(d_{j}\right)}\;\left(4\right)$$

### Disease Semantic Similarity Model II

From above formula (3), it is easy to see that the diseases in the same layer of *DAG*(*D*) will make the same contribution to the semantic value of *D*. Moreover, for diseases in the same layer of *DAG*(*D*), it is reasonable to assume that the diseases appeared in less *DAGs* will be more specific than those diseases appeared in more *DAGs* (Chen et al., 2018a). Hence, in order to protrude the contribution of these more specific diseases, the contribution of the node *d* in *T*(*D*) to the semantic value of the disease *D* could be obtained according to the following formula as well (Chen et al., 2015):

$$D\,2_{D}\,\left(d\right)=-\text{log}\,\left[\frac{\mathrm{the}\,\mathrm{number}\,\mathrm{of}\,D\,A\,G\,s\,\mathrm{containing}\,d}{\mathrm{the}\,\mathrm{number}\,\mathrm{of}\,\mathrm{diseases}}\right]\;\left(5\right)$$

Based on above formula, the semantic value of the disease *D* could be obtained according to the following formula as well:

$$D\,V\,2\,\left(D\right)=\sum_{d\in T\,\left(D\right)}D\,2_{D}\,\left(d\right)\;\left(6\right)$$

Hence, the semantic similarity between two diseases *d*_*i*_ and *d*_*j*_ could be obtained according to the following formula as well:

$$S\,S\,2\,\left(i,j\right)=\frac{\sum_{t\in T\,\left(d_{i}\right)\,\cap T\,\left(d_{j}\right)}\left(D\,2_{d_{i}}\,\left(t\right)+D\,2_{d_{j}}\,\left(t\right)\right)}{D\,V\,2\,\left(d_{i}\right)+D\,V\,2\,\left(d_{j}\right)}\;\left(7\right)$$

### Gaussian Interaction Profile Kernel Similarity for Diseases

According to the assumption that functionally similar miRNAs tend to be more associated with similar diseases, we can further construct the Gaussian interaction profile kernel similarity for diseases by using known miRNA-disease associations. For convenience, let *IP*(*d*_*i*_) denote the *i*th row of the matrix *A*, then the Gaussian interaction profile kernel similarity between two diseases *d*_*i*_ and *d*_*j*_ could be obtained according to the following formula:

$$K\,D\,\left(i,j\right)=\text{exp}\,\left(-\gamma_{d}\,I\,P\,\left(d_{i}\right)-I\,P\,{\left(d_{j}\right)}^{2}\right)\;\left(8\right)$$

Here, the parameter γ_*d*_ is utilized to control the kernel bandwidth and can be obtained through the normalization of the original bandwidth $\gamma_{d}^{{}^{\prime }}$ as follows:

$$\gamma_{d}=\,\frac{\gamma_{d}^{{}^{\prime }}}{(\frac{1}{n}\sum_{i=1}^{n}\left(IP{\left(d_{j}\right)}^{2}\right)}\;\left(9\right)$$

### Gaussian Interaction Profile Kernel Similarity for miRNAs

In a way similar to that of the Gaussian interaction profile kernel similarity for diseases, the Gaussian interaction profile kernel similarity between two miRNAs *m*_*i*_ and *m*_*j*_ could be obtained according to the following formula:

$$K\,M\,\left(i,j\right)=\text{exp}\,\left(-\gamma_{m}\,I\,P\,\left(m_{i}\right)-I\,P\,{\left(m_{j}\right)}^{2}\right)\;\left(10\right)$$

Here, *IP*(*m*_*i*_) denotes the *i*th column of the matrix *A*, and the parameter γ*_*m*_* is utilized to control the kernel bandwidth and can be obtained through the normalization of the original bandwidth $\gamma_{m}^{{}^{\prime }}$ as follows:

$$\gamma_{m}=\frac{\gamma_{m}^{{}^{\prime }}}{(\frac{1}{m}\sum_{i=1}^{m}\left(IP{\left(m_{i}\right)}^{2}\right)}\;\left(11\right)$$

### Integrated Similarity for miRNAs and Diseases

Based on above formulas, for any two diseases *d*_*i*_ and *d*_*j*_, we can obtain an integrated similarity between them according to the following formula:

$$S\,D\,\left(i,j\right)=\left\{ \begin{array}{c} \frac{S\,S\,1\,\left(i,j\right)+S\,S\,2\,\left(i,j\right)}{}\,2\,d_{i}\,\text{and}\,d_{j}\,\mathrm{has}\,\mathrm{semantic}\,\mathrm{similarity}\\ K\,D\,\left(i,j\right)\;\text{otherwise}\;\left(12\right) \end{array}\right.$$

Moreover, in a similar way, for any two miRNAs *m*_*i*_ and *m*_*j*_, we can obtain an integrated similarity between them according to the following formula:

$$S\,M\,\left(i,j\right)=\left\{ \begin{array}{c} F\,S\,\left(i,j\right)\,m_{i}\,\text{and}\,m_{j}\,\mathrm{has}\,\mathrm{functional}\,\mathrm{similarity}\\ K\,M\,\left(i,j\right)\;\text{otherwise}\;\left(13\right) \end{array}\right.$$

### BHCMDA

**FIGURE 2:**
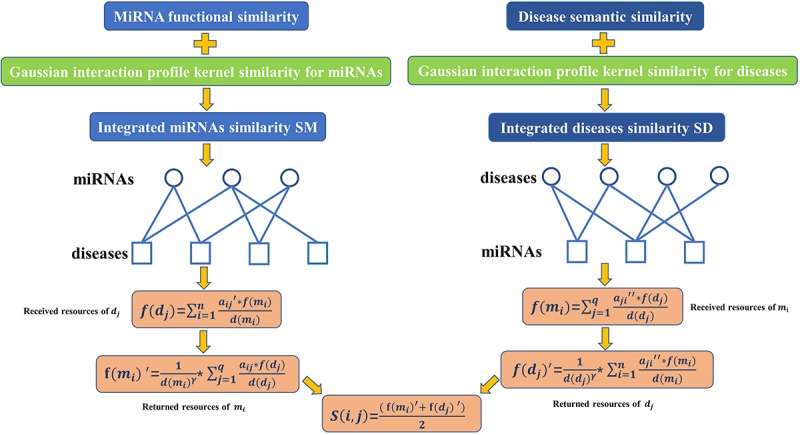
Flow chart of BHCMDA model to predict the potential miRNA-disease associations.

According to the assumption that functionally similar miRNAs are more likely associated with phenotypically similar diseases (Liu et al., 2011), as illustrated in the following Figure 2, we developed a novel computational model called BHCMDA based on the BHC algorithm to predict potential miRNA-disease associations through combining the previously constructed adjacency matrix *A*, the integrated miRNA similarity matrix *SM* and the integrated disease similarity matrix *SD* according to the following steps:

**Step 1:** For convenience, let the *M* = {*m*_1_, *m*_2_, ……*m*_*n*_} and *D* = {*d*_1_, *d*_2_, ……*d*_*q*_} represent all the miRNAs and diseases collected previously, then we can obtain an *n* × *q* dimensional adjacency matrix *A*, an *q* × *q* dimensional integrated diseases similarity matrix *SD*, and an *n* × *n* dimensional integrated miRNAs similarity matrix *SM* according to the above formulas, respectively. Moreover, based on these newly obtained two kinds of matrices such as *A* and *SM*, we can further construct a new *n* × *q* dimensional miRNA-disease association adjacency matrix *A*′ as follows:

$$a_{i\,j}^{{}^{\prime }}=\left\{ \begin{array}{c} 1:\;\text{If}a_{i\,j}=1\;\\ \max_{m_{t}\in M_{i\,j}}SM\left(i,t\right):\;\text{If}\max_{m_{t}\in M_{i\,j}}SM\left(i,t\right)>0\\ 0:\;\text{otherwise}\;\left(14\right) \end{array}\right.$$

**FIGURE 3:**
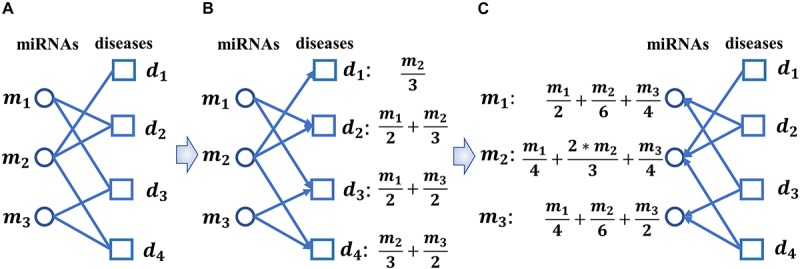
Diagram of implementing the biased heat conduction (BHC) algorithm on the newly constructed bipartite miRNAs-diseases network. **(A)** The newly constructed bipartite miRNAs-diseases network, **(B)** let miRNAs and diseases represent the Object nodes and the User nodes respectively while implementing the BHC algorithm on the newly constructed bipartite miRNAs-diseases network, **(C)** let diseases and miRNAs represent the Object nodes and the User nodes respectively while implementing the BHC algorithm on the newly constructed bipartite miRNAs-diseases network.

Here, *M*_*ij*_ is the set of miRNA nodes that satisfy: ” *m*_*t*_ ∈ *M*_*ij*_, there are *a*_*tj*_ = 1 and *SM*(*i*,*t*) > δ, where δ is a threshold parameter with value between 0 and 1. In this paper, we will set δ = 0.29 according to our simulation results. Thereafter, as illustrated in the following Figure 3A, based on the new adjacency matrix *A*′, we can construct a bipartite miRNAs-diseases network.

**Step 2:** As illustrated in Figure 3B, let miRNAs and diseases represent the Object nodes and the User nodes respectively, then after implementing the BHC algorithm on the newly constructed bipartite miRNAs-diseases network, for any given disease *d*_*j*_ in *D*, the final resources *f*(*d*_*j*_) received by *d*_*j*_ can be obtained according to the following formula while we started from the miRNA nodes:

$$f\,\left(d_{j}\right)=\sum_{i=1}^{n}\frac{a_{i\,j}^{{}^{\prime }}\times f\,\left(m_{i}\right)}{d\,\left(m_{i}\right)}\;\left(15\right)$$

Here, *f*(*m*_*i*_) is the initial resource of the miRNA *m*_*i*_ in *M*, which is set to 1, and *d*(*m*_*i*_) represents the degree of the miRNA node *m*_*i*_ in the newly constructed bipartite miRNAs-diseases network.

**Step 3:** As illustrated in Figure 3C, let diseases and miRNAs represent the Object nodes and the User nodes, respectively, then after implementing the BHC algorithm on the newly constructed bipartite miRNAs-diseases network, for any given miRNA *m*_*i*_ in *M* the final resources *f*(*m*_*i*_)′ received by *m*_*i*_ can be obtained according to the following formula while we started from the disease nodes:

$$f\,{\left(m_{i}\right)}^{\prime }=\frac{1}{d\,{\left(m_{i}\right)}^{\gamma }}\times \,\sum_{j=1}^{q}\frac{a_{i\,j}^{{}^{\prime }}\times f\,\left(d_{j}\right)}{d\,\left(d_{j}\right)}\;\left(16\right)$$

Here, *d*(*d*_*j*_) represents the degree of the disease node *d*_*j*_ in the newly constructed bipartite miRNAs-diseases network, and γ is a parameter to adjust the impact of *d*(*d*_*j*_). In this paper, we set γ = 0.001 according to our simulation results.

**Step 4:** Similar to above step 1, based on these newly constructed two kinds of matrices such as *A* and *SD*, we can also construct another new *n* × *q* dimensional miRNA-disease association adjacency matrix *A*″ as follows:

ai⁢j′′={1:             Ifai⁢j=1          maxdt∈Di⁢jSD(i,t):   Ifmaxdt∈Di⁢jSD(i,t)>00:             otherwise    (17)

Here, *D*_*ij*_ is the set of disease nodes that satisfy: ” *d*_*t*_ ∈ *D*_*ij*_,there are *a*_*jt*_ = 1 and *SD*(*i*,*t*) > η, where η is a threshold parameter with value between 0 and 1. In this paper, we set η = 0.13 according to our simulation results. Thereafter, as illustrated in the following Figure 3A, based on the new adjacency matrix *A*″, we can construct another new bipartite miRNAs-diseases network.

**Step 5:** Similar to above step 2, after implementing the BHC algorithm on the newly constructed bipartite miRNAs-diseases network, for any given miRNA *m*_*i*_ in *M*, the final resources *f*(*m*_*i*_)″ received by *m*_*i*_ can be obtained according to the following formula while we started from the disease nodes:

f⁢(mi)″=∑j=1qaj⁢i′′×f⁢(dj)′d⁢(dj)        (18)

Here, *f*(*d*_*j*_)′ is the initial resource of the disease *d*_*j*_ in *D*, which is set to 1, and *d*(*d*_*j*_) represents the degree of the disease node *d*_*j*_ in the newly constructed bipartite miRNAs-diseases network.

**Step 6:** Similar to above step 3, after implementing the BHC algorithm on the newly constructed bipartite miRNAs-diseases network, for any given

disease *d*_*j*_ in *D*, the final resources *f*(*d*_*j*_)″ received by *d*_*j*_ can be obtained according to the following formula while we started from the miRNA nodes:

f⁢(dj)″=1d⁢(dj)γ×⁢∑i=1naj⁢i′′×f⁢(mi)′′d⁢(mi)     (19)

Here, *d*(*m*_*i*_) represents the degree of the miRNA node *m*_*i*_ in the newly constructed bipartite miRNAs-diseases network, and γ is a parameter to adjust the impact of *d*(*d*_*j*_). In this paper, we set γ = 0.001 according to our simulation results.

**Step 7:** Finally, based on above formulas, the association score between miRNA *m*_*i*_ and disease *d*_*j*_ can be calculated as follows:

$$S\,\left(i,j\right)=\frac{\left(\,f\,{\left(m_{i}\right)}^{{}^{\prime }}+\,f\,\left(d_{j}\right){\,}^{\prime \prime }\right)}{2}\;\left(20\right)$$

## Results

### Performance Evaluation

In order to evaluate the predictive performance of BHCMDA, twofold cross-validation, fivefold cross-validation and LOOCV were implemented separately based on the known miRNA-disease associations downloaded from the HMDD V2.0 database. In LOOCV, every known miRNA-disease association takes turns to act as the test sample and the rest of known miRNA-disease associations serve as training samples. Moreover, all these miRNA-disease pairs having no known associations play the role of candidate samples, then we can obtain the ranking of each test sample with all candidate samples according to their predicted scores after implementing BHCMDA. If the rank of the test sample is higher than the given threshold, it will be considered as a correct prediction. In the framework of fivefold cross-validation, all known miRNA-disease associations are randomly divided into five equal groups without overlap first, then each group acts as test samples in turn and the other four groups serve as training samples. Besides, all these miRNA-disease pairs having no known associations play the role of candidate samples. After the scores of candidate samples and the test samples have been calculated, we take turns to compare the score of each test sample with the scores of candidate samples. If the rank of the test sample exceeds the given threshold, it will be thought as a successful prediction. Furthermore, the receiver-operating characteristics (ROC) curve can be painted to assess the performance of BHCMDA by computing false positive rate (FPR, 1-specificity) and true positive rate (TPR, sensitivity) on the basis of varying thresholds (Le et al., 2019). Here, sensitivity means the percentage of positive test samples whose rankings exceed the given threshold, while 1-specificity denotes the percentage of candidate samples with rankings under the given threshold. Then, area under the ROC curves (AUCs) can be calculated to evaluate the predictive performance of BHCMDA, the larger the value, the better the prediction performance of BHCMDA.

**FIGURE 4:**
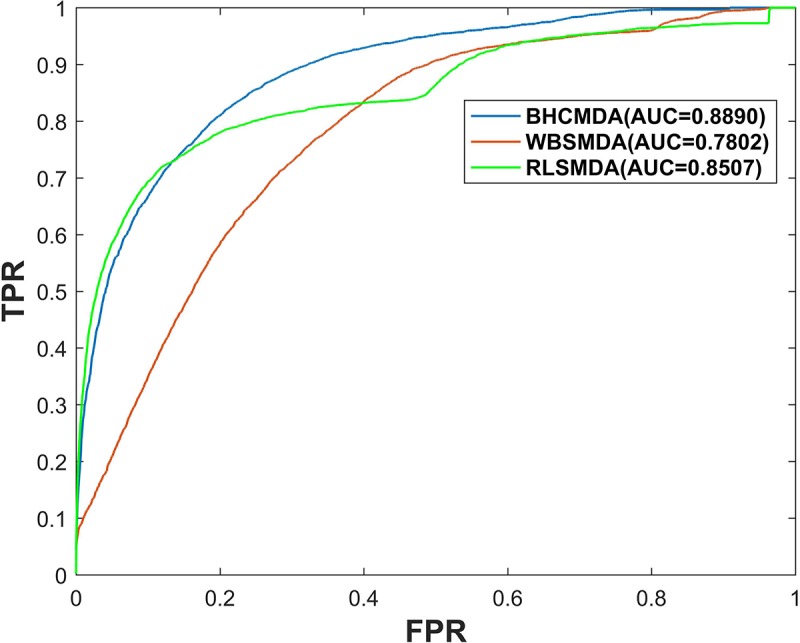
Performance comparisons between BHCMDA, LRLSLDA, and WBSMDA in LOOCV.

**FIGURE 5:**
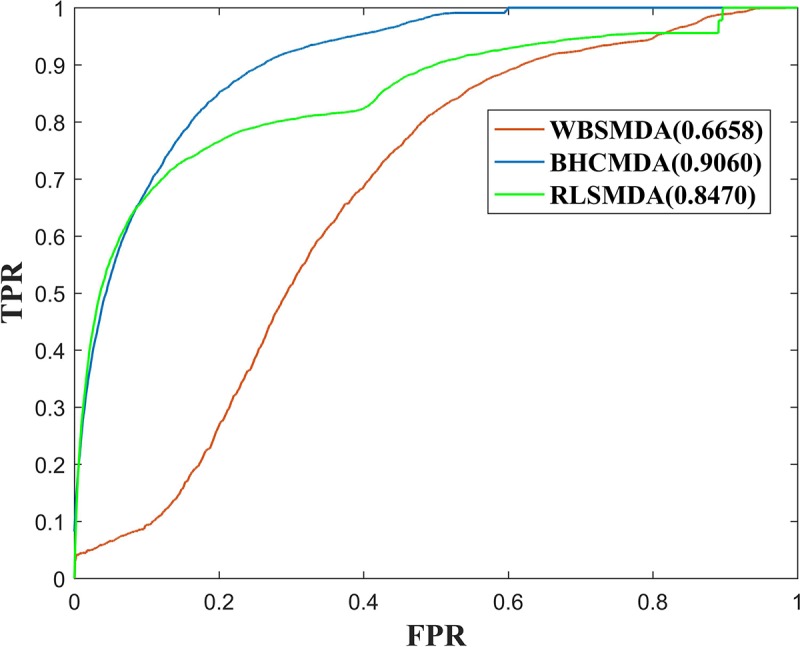
Performance comparisons between BHCMDA, LRLSLDA, and WBSMDA in twofold cross-validation.

**FIGURE 6:**
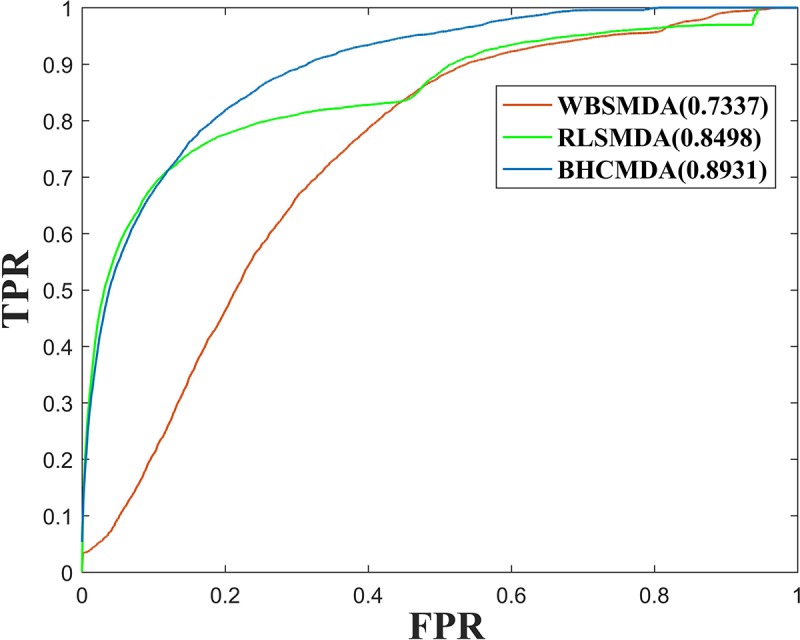
Performance comparisons between BHCMDA, LRLSLDA, and WBSMDA in fivefold cross-validation.

As a result, BHCMDA can achieve reliable AUCs of 0.8890, 0.9060, and 0.8931 under the frameworks of global LOOCV, twofold cross-validation and fivefold cross-validation respectively. Moreover, we compared BHCMDA with two kinds of state-of-the-art models such as RLSMDA (Chen and Yan, 2014) and WBSMDA (Chen et al., 2016b). As illustrated in the Figure 4, RLSMDA and WBSMDA can achieve AUCs of 0.8507 and 0.7802 under the frameworks of global LOOCV respectively, which are inferior to the BHCMDA’s AUCs. Besides, as shown in the Figure 5, under the twofold cross-validation framework, the AUCs of RLSMDA and WBSMDA are 0.8470 and 0.6658 respectively, indicating that the AUCs of BHCMDA is higher than RLSMDA and WBSMDA. What’s more, as illustrated in the Figure 6, RLSMDA and WBSMDA can achieve AUCs of 0.8498 and 0.7337 under the frameworks of fivefold cross-validation respectively, which are also lower than the BHCMDAs’ AUCs. In conclusion, it is obvious that BHCMDA has better performance than RLSMDA and WBSMDA in miRNA-disease association prediction.

### Case Studies

In order to further assess the predictive performance of BHCMDA, we conducted case studies of three kinds of human diseases such as esophageal neoplasms, colonic neoplasms and lymphoma, and the predicted results were verified by evidences illustrated in HMDD v3.0^2^, dbDEMC 2.0^3^, dbDEMC (Yang et al., 2010) and miR2Disease (Jiang et al., 2008), respectively.

Esophageal neoplasms is the eighth common cancer in the world according to the pathological characteristics (He et al., 2012). As the tumor grows, the patient may suffer from difficult or painful swallowing, coughing up blood and weight loss. The number of men having esophageal cancer are three to four times than that of women, and the survival rates are low (Enzinger and Mayer, 2003). The main treatment for esophageal neoplasms is cisplatin-based chemotherapy, but the chemotherapy reaction is difficult to detect. Therefore, the earlier the esophageal tumor is found, the more helpful it will be in the cancer treatment (Xie et al., 2013; Wan et al., 2016). A large number of miRNAs have been confirmed to be associated with esophageal neoplasms. For instance, the overexpression of hsa-miR-17 cluster can promote the growth of esophageal tumor cell. In addition, hsa-let-7 can server as the prognostic biomarker for weighing the response to chemotherapy (Liao et al., 2014; Xu et al., 2014). While implementing BHCMDA to predict associated miRNAs of esophageal neoplasms, there are 9 out of the top-10 and 44 out of the top-50 predicted miRNAs having been verified to be related with esophageal neoplasms according to confirmations provided by dbDEMC and dbDEMC 2.0, respectively (see Table 1).

**TABLE 1:** Top 50 potential Esophageal Neoplasms-related miRNAs predicted by BHCMDA and confirmations for these predicted associations provided by the dbDEMC and dbDEMC 2.0.

**miRNA**	**Evidence**	**miRNA**	**Evidence**
hsa-mir-17	dbDEMC	hsa-mir-302c	dbDEMC
hsa-mir-18a	dbDEMC 2.0	hsa-mir-602	dbDEMC
hsa-mir-200b	dbDEMC	hsa-mir-612	dbDEMC 2.0
hsa-mir-629	dbDEMC 2.0	hsa-mir-657	unconfirmed
hsa-mir-93	dbDEMC 2.0	hsa-mir-376c	dbDEMC 2.0
hsa-mir-324	dbDEMC	hsa-mir-367	dbDEMC 2.0
hsa-mir-19b	dbDEMC 2.0	hsa-mir-153	dbDEMC
hsa-let-7d	dbDEMC	hsa-mir-302e	dbDEMC
hsa-mir-185	dbDEMC 2.0	hsa-mir-30c	dbDEMC 2.0
hsa-mir-638	unconfirmed	hsa-mir-302d	dbDEMC 2.0
hsa-let-7f	dbDEMC 2.0	hsa-mir-16	dbdemc 2.0
hsa-mir-601	unconfirmed	hsa-mir-429	dbDEMC 2.0
hsa-mir-1	dbDEMC 2.0	hsa-mir-106b	dbDEMC 2.0
hsa-let-7i	dbDEMC 2.0	hsa-mir-583	dbDEMC
hsa-let-7e	dbDEMC	hsa-mir-125b	dbDEMC 2.0
hsa-let-7g	dbDEMC	hsa-mir-660	dbDEMC
hsa-mir-637	dbDEMC 2.0	hsa-mir-557	dbDEMC 2.0
hsa-mir-218	dbDEMC 2.0	hsa-mir-600	unconfirmed
hsa-mir-608	unconfirmed	hsa-mir-611	unconfirmed
hsa-mir-596	dbDEMC 2.0	hsa-mir-654	dbDEMC 2.0
hsa-mir-615	dbDEMC	hsa-mir-662	dbDEMC 2.0
hsa-mir-622	dbDEMC	hsa-mir-769	dbDEMC
hsa-mir-518c	dbDEMC 2.0	hsa-mir-215	dbDEMC 2.0
hsa-mir-301a	HMDD3.0	hsa-mir-335	dbDEMC 2.0
hsa-mir-302b	dbDEMC	hsa-mir-221	dbDEMC 2.0

Colonic neoplasms is a common malignant tumor which poses a huge threat to human lives in the world (Jemal et al., 2011; Ogata-Kawata et al., 2014). It is reported that about half of colonic neoplasms patients may die of metastatic disease in five years from diagnosis (Parkin et al., 2005; Drusco et al., 2014). Therefore, early diagnosis of colon cancer is of great significance in improving the patients’ survival rate. In the recent years, investigators have verified a few miRNAs related with colonic neoplasms. Take Mir-199a-3p (the 3p arm of the pre-miRNA for miR-199a) as an example, it is highly expressed in colonic neoplasms tissues, resulting in significantly reduced survival rate of patients (Wan et al., 2013). In addition, tumor specimens illustrated highly significant and large multiple differential expressions of levels of some miRNAs, including mir-1, mir-31, mir-133a, mir-135b and others (Sarver et al., 2009). While implementing BHCMDA to discern the potentially relevant miRNAs of colonic neoplasms, there are 8 out of the top-10 and 46 out of the top-50 predicted miRNAs having been validated to be related with colonic neoplasms by confirmations provided by dbDEMC, dbDEMC 2.0, HMDD3.0 and miR2Disease, respectively (see Table 2).

**TABLE 2:** Top 50 potential Colonic Neoplasms-related miRNAs predicted by BHCMDA and confirmations for these predicted associations provided by the dbDEMC, dbDEMC 2.0, HMDD3.0 and miR2Disease.

**miRNA**	**Evidence**	**miRNA**	**Evidence**
hsa-mir-324	unconfirmed	hsa-mir-146b	dbDEMC 2.0
hsa-mir-222	dbDEMC 2.0	hsa-mir-601	dbDEMC 2.0
hsa-mir-301a	dbDEMC 2.0	hsa-mir-7	dbDEMC 2.0
hsa-mir-638	dbDEMC 2.0	hsa-mir-637	dbDEMC 2.0
hsa-mir-200a	unconfirmed	hsa-mir-526a	dbDEMC 2.0
hsa-mir-210	dbDEMC 2.0	hsa-mir-515	unconfirmed
hsa-mir-133a	dbDEMC 2.0	hsa-mir-27a	dbDEMC 2.0
hsa-mir-93	dbDEMC 2.0	hsa-mir-331	HMDD3.0
hsa-mir-185	dbDEMC 2.0	hsa-mir-148a	dbDEMC 2.0
hsa-mir-367	dbDEMC 2.0	hsa-mir-195	dbDEMC 2.0
hsa-mir-219	unconfirmed	hsa-mir-520h	dbDEMC 2.0
hsa-mir-520a	HMDD3.0	hsa-mir-153	dbDEMC 2.0
hsa-mir-196a	dbDEMC 2.0	hsa-mir-199b	dbDEMC 2.0
hsa-mir-199a	23292866	hsa-mir-30b	dbDEMC 2.0
hsa-mir-297	dbDEMC 2.0	hsa-mir-26a	dbDEMC
hsa-mir-608	dbDEMC 2.0	hsa-mir-181b	dbDEMC 2.0
hsa-mir-449b	dbDEMC 2.0	hsa-mir-520e	dbDEMC 2.0
hsa-mir-34c	miR2Disease	hsa-mir-602	dbDEMC 2.0
hsa-mir-215	dbDEMC 2.0	hsa-mir-512	HMDD3.0
hsa-mir-375	dbDEMC 2.0	hsa-mir-194	dbDEMC 2.0
hsa-mir-25	dbDEMC 2.0	hsa-mir-95	dbDEMC 2.0
hsa-mir-34b	dbDEMC	hsa-mir-612	dbDEMC 2.0
hsa-mir-429	dbDEMC 2.0	hsa-mir-526b	dbDEMC 2.0
hsa-mir-203	dbDEMC 2.0	hsa-mir-657	dbDEMC 2.0
hsa-mir-518b	dbDEMC 2.0	hsa-mir-135a	dbDEMC 2.0

There are two types of lymphoma, one is Hodgkin Lymphomas (HL) and the other is non-Hodgkin Lymphomas (NHL). HL is a more common form of lymphoma and it is difficult to be diagnosed at an early stage (Coiffier, 2006; Xie et al., 2012). NHL is a heterogeneous malignant tumor originating from lymphoid hematopoietic tissue and it is mainly treated by local radiotherapy and chemotherapy (Coiffier, 2006). An example of miRNAs related with lymphoma is miR-125b. By inhibiting miR-125b-5p (The 5p arm of the pre-miRNA for mir-125b), lymphoma cells will be sensitive to anticancer drugs such as bortezomib (Manfè et al., 2013). Besides, the overexpressed miR-142-5p (the 5p arm of the pre-miRNA for miR-142) which was found in gastric MALT lymphoma played a vital role in the pathogenesis of this cancer (Saito et al., 2012). Furthermore, the upregulation of miRNA hsa-mir-9, hsa-mir-34a, hsa-mir-183, hsa-mir-215 and down-regulation of hsa-mir-30b were all relevant to lymphoma’s development based on experimental literatures. While implementing BHCMDA to infer the potentially relevant miRNAs of Lymphoma, there are 10 out of the top-10 and 46 out of the top-50 predicted miRNAs having been confirmed to be associated with Lymphomas by confirmations provided by dbDEMC 2.0 and the recent experimental literatures with relevant PMIDs, respectively (see Table 3).

**TABLE 3:** Top 50 potential Lymphomas-related miRNAs predicted by BHCMDA and confirmations for these predicted associations provided by the dbDEMC 2.0 and the recent experimental literatures with relevant PMIDs.

**miRNA**	**Evidence**	**disease**	**Evidence**
hsa-mir-145	dbDEMC 2.0	hsa-mir-652	dbDEMC 2.0
hsa-mir-34a	dbDEMC 2.0	hsa-mir-221	dbDEMC 2.0
hsa-mir-29b	dbDEMC 2.0	hsa-mir-185	dbDEMC 2.0
hsa-mir-9	dbDEMC 2.0	hsa-mir-596	dbDEMC 2.0
hsa-mir-106b	dbDEMC 2.0	hsa-mir-608	dbDEMC 2.0
hsa-let-7a	dbDEMC 2.0	hsa-mir-223	dbDEMC 2.0
hsa-mir-125b	dbDEMC 2.0	hsa-mir-557	dbDEMC 2.0
hsa-mir-183	dbDEMC 2.0	hsa-mir-192	dbDEMC 2.0
hsa-mir-205	dbDEMC 2.0	hsa-mir-602	dbDEMC 2.0
hsa-mir-30b	dbDEMC 2.0	hsa-mir-181b	dbDEMC 2.0
hsa-mir-29a	dbDEMC 2.0	hsa-mir-214	dbDEMC 2.0
hsa-mir-93	dbDEMC 2.0	hsa-let-7c	dbDEMC 2.0
hsa-mir-199a	dbDEMC 2.0	hsa-let-7i	dbDEMC 2.0
hsa-mir-324	unconfirmed	hsa-mir-612	unconfirmed
hsa-mir-143	dbDEMC 2.0	hsa-mir-657	dbDEMC 2.0
hsa-mir-106a	dbDEMC 2.0	hsa-mir-142	23209550
hsa-let-7b	dbDEMC 2.0	hsa-mir-222	dbDEMC 2.0
hsa-mir-30e	dbDEMC 2.0	hsa-let-7d	dbDEMC 2.0
hsa-mir-638	dbDEMC 2.0	hsa-mir-153	dbDEMC 2.0
hsa-mir-215	dbDEMC 2.0	hsa-mir-367	dbDEMC 2.0
hsa-mir-637	dbDEMC 2.0	hsa-mir-518c	unconfirmed
hsa-mir-195	dbDEMC 2.0	hsa-mir-622	dbDEMC 2.0
hsa-mir-598	dbDEMC 2.0	hsa-mir-583	dbDEMC 2.0
hsa-let-7e	dbDEMC 2.0	hsa-mir-600	dbDEMC 2.0
hsa-mir-615	unconfirmed	hsa-mir-601	dbDEMC 2.0

## Discussion

In recent years, a growing number of computational models have been proposed to find underlying miRNA-disease associations. In this article, we put forward a prediction model called BHCMDA based on the BHC algorithm to discover potential associated miRNAs of the diseases by integrating known miRNA-disease associations, the disease semantic similarity, the miRNA functional similarity, and the Gaussian interaction profile kernel similarity. In order to estimate the prediction performance of BHCMDA, LOOCV, twofold cross-validation and fivefold cross-validation were implemented, respectively. Moreover, three different kinds of case studies were conducted as well. Simulation results from both case studies and cross-validations demonstrated that BHCMDA had splendid performance in prediction of potential miRNA-disease associations.

There are a few reasons to explain the reliable performance of BHCMDA. In the first place, the data used to predict potential miRNA-disease associations obtained from HMDD V2.0 in this model is rich and reliable. In addition, BHCMDA not only integrates the disease semantic similarity and the miRNA functional similarity with the Gaussian interaction profile kernel similarity, but also applies a clustering algorithm based on the integrated data, which makes the basic data richer and more accurate. In the end, BHC algorithm has the ability to recommend unpopular products. We averaged the predicted data obtained by using BHC algorithm, which made the prediction more reliable.

Whereas there still exist some limitations in BHCMDA. For instance, the quantity of known miRNA-disease associations is still not adequate. In addition, we developed BHCMDA according to the assumption that functionally similar miRNAs are more likely associated with phenotypically similar diseases, which may bring about bias to miRNAs related with more known diseases. Obviously, all these limitations in BHCMDA deserve further study and need to be improved in the future.

## Data Availability Statement

Generated Statement: Publicly available datasets were analyzed in this study. These data can be found here: HMDD database (http://www.cuilab.cn/hmdd), miRNA functional similarity (http://www.cuilab.cn/files/images/cuilab/misim.zip), Mesh database (http://www.ncbi.nlm.nih.gov/),dbDEMC database (doi: 10.1186/1471-2164-11-s4-s5), dbDEMC 2.0 (http://www.picb.ac.cn/dbDEMC), HMDD 3.0 (http://www.cuilab.cn/hmdd), miR2Disease (doi: 10.1093/nar/gkn714).

## Author Contributions

XW and XZ conceived the study. XW, LK, and HZ improved the study based on the original model. XZ and TP implemented the algorithms corresponding to the study. LW, XZ, and LK supervised the study. XW and LW wrote the manuscript. All authors reviewed and improved the manuscript.

## Conflict of Interest

The authors declare that the research was conducted in the absence of any commercial or financial relationships that could be construed as a potential conflict of interest.

## Footnotes

1 http://www.ncbi.nlm.nih.gov/

2 http://www.cuilab.cn/hmdd

3 http://www.picb.ac.cn/dbDEMC

## References

[B1] Bartel D. P. (2009). MicroRNAs: target recognition and regulatory functions. *Cell* 136 215–233. 10.1016/j.cell.2009.01.002 19167326 PMC3794896

[B2] Chen X. (2015). KATZLDA: KATZ measure for the lncRNA-disease association prediction. *Sci. Rep.* 5:16840 10.1038/srep16840 PMC4649494 26577439

[B3] Chen X. Clarence Yan C. Luo C. Ji W. Zhang Y. Dai Q. (2015). Constructing lncRNA functional similarity network based on lncRNA-disease associations and disease semantic similarity. *Sci. Rep.* 5:11338 10.1038/srep11338 PMC4462156 26061969

[B4] Chen X. Guan N. N. Li J. Q. Yan G. Y. (2018a). GIMDA: Graphlet interaction-based MiRNA-disease association prediction. *J. Cell Mol. Med.* 22 1548–1561. 10.1111/jcmm.13429 29272076 PMC5824414

[B5] Chen X. Huang L. Xie D. Zhao Q. (2018b). EGBMMDA: extreme gradient boosting machine for MiRNA-disease association prediction. *Cell Death Dis.* 9:3 10.1038/s41419-017-0003-x PMC5849212 29305594

[B6] Chen X. Huang Y.-A. Wang X.-S. You Z.-H. Chan K. C. C. (2016a). FMLNCSIM: fuzzy measure-based lncRNA functional similarity calculation model. *Oncotarget* 7 45948–45958. 10.18632/oncotarget.10008 27322210 PMC5216773

[B7] Chen X. Yan C. C. Zhang X. You Z.-H. Deng L. Liu Y. (2016b). WBSMDA: within and between score for miRNA-disease association prediction. *Sci. Rep.* 6:21106 10.1038/srep21106 PMC4754743 26880032

[B8] Chen X. Liu M. X. Yan G. Y. (2012). RWRMDA: predicting novel human microRNA-disease associations. *Mol. Biosyst.* 8 2792–2798. 10.1039/c2mb25180a 22875290

[B9] Chen X. Yan G. Y. (2014). Semi-supervised learning for potential human microRNA-disease associations inference. *Sci. Rep.* 4:5501 10.1038/srep05501 PMC4074792 24975600

[B10] Cheng A. M. Byrom M. W. Shelton J. Ford L. P. (2005). Antisense inhibition of human miRNAs and indications for an involvement of miRNA in cell growth and apoptosis. *Nucleic Acids Res.* 33 1290–1297. 10.1093/nar/gki200 15741182 PMC552951

[B11] Coiffier B. (2006). Monoclonal antibody as therapy for malignant lymphomas. *C. R. Biol.* 329 241–254. 10.1016/j.crvi.2005.12.006 16644494

[B12] Cui Q. Yu Z. Purisima E. O. Wang E. (2006). Principles of microRNA regulation of a human cellular signaling network. *Mol. Syst. Biol.* 2:46 10.1038/msb4100089 PMC1681519 16969338

[B13] Drusco A. Nuovo G. J. Zanesi N. Di Leva G. Pichiorri F. Volinia S. (2014). MicroRNA profiles discriminate among colon cancer metastasis. *PLoS One* 9:e96670 10.1371/journal.pone.0096670 PMC4055753 24921248

[B14] Enzinger P. C. Mayer R. J. (2003). Esophageal cancer. *New Engl. J. Med.* 349 2241–2252. 10.1056/NEJMra035010 14657432

[B15] He B. Yin B. Wang B. Xia Z. Chen C. Tang J. (2012). MicroRNAs in esophageal cancer (review). *Mol. Med. Rep.* 6 459–465. 10.3892/mmr.2012.975 22751839

[B16] Hirota T. Date Y. Nishibatake Y. Takane H. Fukuoka Y. Taniguchi Y. (2012). Dihydropyrimidine dehydrogenase (DPD) expression is negatively regulated by certain microRNAs in human lung tissues. *Lung Cancer* 77 16–23. 10.1016/j.lungcan.2011.12.018 22306127

[B17] Huang Y.-A. Chen X. You Z.-H. Huang D.-S. Chan K. C. C. (2016). ILNCSIM: improved lncRNA functional similarity calculation model. *Oncotarget* 7 25902–25914. 10.18632/oncotarget.8296 27028993 PMC5041953

[B18] Jemal A. Bray F. Center M. M. Ferlay J. Ward E. Forman D. (2011). Global cancer statistics. *CA Cancer J. Clin.* 61 69–90. 10.3322/caac.20107 21296855

[B19] Jiang Q. Hao Y. Wang G. Juan L. Zhang T. Teng M. (2010). Prioritization of disease microRNAs through a human phenome-microRNAome network. *BMC Syst. Biol.* 4:S2 10.1186/1752-0509-4-S1-S2 PMC2880408 20522252

[B20] Jiang Q. Wang Y. Hao Y. Juan L. Teng M. Zhang X. (2008). miR2Disease: a manually curated database for microRNA deregulation in human disease. *Nucleic Acids Res.* 37(Suppl._1), D98–D104. 10.1093/nar/gkn714 18927107 PMC2686559

[B21] Jopling C. L. Yi M. Lancaster A. M. Lemon S. M. Sarnow P. (2005). Modulation of hepatitis C virus RNA abundance by a liver-specific MicroRNA. *Science* 309 1577–1581. 10.1126/science.1113329 16141076

[B22] Le N. Q. Ho Q. T. Ou Y. Y. (2017). Incorporating deep learning with convolutional neural networks and position specific scoring matrices for identifying electron transport proteins. *J. Comput. Chem.* 38 2000–2006. 10.1002/jcc.24842 28643394

[B23] Le N. Q. Ho Q. T. Ou Y. Y. (2018). Classifying the molecular functions of Rab GTPases in membrane trafficking using deep convolutional neural networks. *Anal. Biochem.* 555 33–41. 10.1016/j.ab.2018.06.011 29908156

[B24] Le N. Q. K. Yapp E. K. Y. Ho Q. T. Nagasundaram N. Ou Y. Y. Yeh H. Y. (2019). iEnhancer-5Step: identifying enhancers using hidden information of DNA sequences via Chou’s 5-step rule and word embedding. *Anal. Biochem.* 571 53–61. 10.1016/j.ab.2019.02.017 30822398

[B25] Li Y. Qiu C. Tu J. Geng B. Yang J. Jiang T. (2013). HMDD v2.0: a database for experimentally supported human microRNA and disease associations. *Nucleic Acids Res.* 42 D1070–D1074. 10.1093/nar/gkt1023 24194601 PMC3964961

[B26] Liao J. Liu R. Yin L. Pu Y. (2014). Expression profiling of exosomal miRNAs derived from human esophageal cancer cells by solexa high-throughput sequencing. *Intern. J. Mol. Sci.* 15:15530 10.3390/ijms150915530 PMC4200790 25184951

[B27] Liu J.-G. Zhou T. Guo Q. (2011). Information filtering via biased heat conduction. *Phys. Rev. E* 84:037101 10.1103/PhysRevE.84.037101 22060533

[B28] Luo J. Xiao Q. Liang C. Ding P. (2017). Predicting microRNA-disease associations using kronecker regularized least squares based on heterogeneous omics data. *IEEE Access* 5 2503–2513. 10.1109/ACCESS.2017.2672600

[B29] Manfè V. Biskup E. Willumsgaard A. Skov A. G. Palmieri D. Gasparini P. (2013). cMyc/miR-125b-5p signalling determines sensitivity to bortezomib in preclinical model of cutaneous T-cell lymphomas. *PLoS One* 8:e59390 10.1371/journal.pone.0059390 PMC3602111 23527180

[B30] Meister G. Tuschl T. (2004). Mechanisms of gene silencing by double-stranded RNA. *Nature* 431 343–349. 10.1038/nature02873 15372041

[B31] Miska E. A. (2005). How microRNAs control cell division, differentiation and death. *Curr. Opin. Genet. Dev.* 15 563–568. 10.1016/j.gde.2005.08.005 16099643

[B32] Mork S. Pletscher-Frankild S. Palleja Caro A. Gorodkin J. Jensen L. J. (2014). Protein-driven inference of miRNA-disease associations. *Bioinformatics* 30 392–397. 10.1093/bioinformatics/btt677 24273243 PMC3904518

[B33] Ogata-Kawata H. Izumiya M. Kurioka D. Honma Y. Yamada Y. Furuta K. (2014). Circulating exosomal microRNAs as biomarkers of colon cancer. *PLoS One* 9:e92921 10.1371/journal.pone.0092921 PMC3976275 24705249

[B34] Paraskevi A. Theodoropoulos G. Papaconstantinou I. Mantzaris G. Nikiteas N. Gazouli M. (2012). Circulating MicroRNA in inflammatory bowel disease. *J. Crohns. Colitis* 6 900–904. 10.1016/j.crohns.2012.02.006 22386737

[B35] Parkin D. M. Bray F. Ferlay J. Pisani P. (2005). Global cancer statistics, 2002. *CA Cancer J. Clin.* 55 74–108. 10.3322/canjclin.55.2.74 15761078

[B36] Png K. J. Yoshida M. Zhang X. H. Shu W. Lee H. Rimner A. (2011). MicroRNA-335 inhibits tumor reinitiation and is silenced through genetic and epigenetic mechanisms in human breast cancer. *Genes Dev.* 25 226–231. 10.1101/gad.1974211 21289068 PMC3034897

[B37] Saito Y. Suzuki H. Tsugawa H. Imaeda H. Matsuzaki J. Hirata K. (2012). Overexpression of miR-142-5p and miR-155 in gastric mucosa-associated lymphoid tissue (MALT) lymphoma resistant to *Helicobacter* pylori eradication. *PLoS One* 7:e47396 10.1371/journal.pone.0047396 PMC3509063 23209550

[B38] Sarver A. L. French A. J. Borralho P. M. Thayanithy V. Oberg A. L. Silverstein K. A. T. (2009). Human colon cancer profiles show differential microRNA expression depending on mismatch repair status and are characteristic of undifferentiated proliferative states. *BMC Cancer* 9:401 10.1186/1471-2407-9-401 PMC2787532 19922656

[B39] Shi H. Xu J. Zhang G. Xu L. Li C. Wang L. (2013). Walking the interactome to identify human miRNA-disease associations through the functional link between miRNA targets and disease genes. *BMC Syst. Biol.* 7:101 10.1186/1752-0509-7-101 PMC4124764 24103777

[B40] Tavazoie S. F. Alarcon C. Oskarsson T. Padua D. Wang Q. Bos P. D. (2008). Endogenous human microRNAs that suppress breast cancer metastasis. *Nature* 451 147–152. 10.1038/nature06487 18185580 PMC2782491

[B41] Valastyan S. Reinhardt F. Benaich N. Calogrias D. Szasz A. M. Wang Z. C. (2009). A pleiotropically acting microRNA, miR-31, inhibits breast cancer metastasis. *Cell* 137 1032–1046. 10.1016/j.cell.2009.03.047 19524507 PMC2766609

[B42] Wan D. He S. Xie B. Xu G. Gu W. Shen C. (2013). Aberrant expression of miR-199a-3p and its clinical significance in colorectal cancers. *Med. Oncol.* 30:378. 10.1007/s12032-012-0378-6 23292866

[B43] Wan J. Wu W. Che Y. Kang N. Zhang R. (2016). Insights into the potential use of microRNAs as a novel class of biomarkers in esophageal cancer. *Dis. Esophagus.* 29 412–420. 10.1111/dote.12338 25789723

[B44] Wang B. Wang H. Yang Z. (2012). MiR-122 inhibits cell proliferation and tumorigenesis of breast cancer by targeting IGF1R. *PLoS One* 7:e47053 10.1371/journal.pone.0047053 PMC3466252 23056576

[B45] Wang D. Wang J. Lu M. Song F. Cui Q. (2010). Inferring the human microRNA functional similarity and functional network based on microRNA-associated diseases. *Bioinformatics* 26 1644–1650. 10.1093/bioinformatics/btq241 20439255

[B46] Xie L. Ushmorov A. Leithäuser F. Guan H. Steidl C. Färbinger J. (2012). FOXO1 is a tumor suppressor in classical Hodgkin lymphoma. *Blood* 119 3503–3511. 10.1182/blood-2011-09-381905 22343918

[B47] Xie Z. Chen G. Zhang X. Li D. Huang J. Yang C. (2013). Salivary microRNAs as promising biomarkers for detection of esophageal cancer. *PLoS One* 8:e57502 10.1371/journal.pone.0057502 PMC3613402 23560033

[B48] Xu P. Guo M. Hay B. A. (2004). MicroRNAs and the regulation of cell death. *Trends Genet.* 20 617–624. 10.1016/j.tig.2004.09.010 15522457

[B49] Xu X.-L. Jiang Y.-H. Feng J.-G. Su D. Chen P.-C. Mao W.-M. (2014). MicroRNA-17, microRNA-18a, and microRNA-19a are prognostic indicators in esophageal squamous cell carcinoma. *Ann. Thorac. Surg.* 97 1037–1045. 10.1016/j.athoracsur.2013.10.042 24360091

[B50] Xuan P. Han K. Guo M. Guo Y. Li J. Ding J. (2013). Prediction of microRNAs associated with human diseases based on weighted k most similar neighbors. *PLoS One* 8:e70204 10.1371/journal.pone.0070204 PMC3738541 23950912

[B51] Yang Z. Ren F. Liu C. He S. Sun G. Gao Q. (2010). dbDEMC: a database of differentially expressed miRNAs in human cancers. *BMC Genomics* 11(Suppl. 4):S5. 10.1186/1471-2164-11-S4-S5 PMC3005935 21143814

[B52] Zou Q. Li J. Hong Q. Lin Z. Wu Y. Shi H. (2015). Prediction of microRNA-disease associations based on social network analysis methods. *Biomed. Res. Int.* 2015:810514 10.1155/2015/810514 PMC4529919 26273645

